# Systemic mastocytosis with an associated hematological neoplasms: One or two entities?

**DOI:** 10.1002/jha2.22

**Published:** 2020-06-04

**Authors:** Linet Njue, Naomi Porret, Michaela Fux, Ulrike Bacher, Yara Banz, Alicia Rovó

**Affiliations:** ^1^ Department of Hematology and Central Hematology Laboratory, Inselspital, Bern University Hospital University of Bern Bern Switzerland; ^2^ University Institute of Clinical Chemistry, Inselspital, Bern University Hospital University of Bern Bern Switzerland; ^3^ Institute of Pathology University of Bern Bern Switzerland

Mastocytosis refers to a group of disorders characterized by excessive mast‐cell accumulation in one or multiple tissues. The WHO classification [1] splits mastocytosis from the myeloproliferative neoplasms as a distinctive entity group. SM‐AHN was introduced as abbreviation name of the 2008 category of “systemic mastocytosis with associated clonal hematological non‐mast‐cell lineage disease" (SH‐AHNMD) and recognized as a subgroup of systemic mastocytosis. Approximately 5% of SM cases are associated with myeloid malignancies, according to the WHO classification, the most commonly detected AHN is CMML [1]. Here, we report a case with the diagnosis of SM‐AHN combining SM and CMML. We aim to show that SM‐AHN with CMML may be overestimated, and that monocytosis may be an integral component of the SM.

A 62‐year‐old caucasian man was referred to our outpatient clinic in July 2018 with suspicion of myeloid disease. The patient complained about episodes of extreme fatigue, drenching night sweats, fever, skin hematomas, and weight loss of about 8 kg within 6 months. The initial blood examinations documented a hemoglobin level of 10.5 g/dL, platelet count of 98 × 10^9^/L, and white blood cell count of 13.5 × 10^9^/L showing a differential count with 8.47 × 10^9^/L neutrophils, 0.98 × 10^9^/L lymphocytes, and 2.29 × 10^9^/L monocytes (17% of total leukocytes), no eosinophilia or basophilia. Serum tryptase level was increased at 139 µg/L (normal < 13 µg/L) with a normal serum lactate dehydrogenase of 313 U/L. A full‐body computed‐tomography scan revealed homogenous splenomegaly with a bipolar diameter of 18.5 cm.

The morphological examination of the initial bone marrow (BM) aspirate and trephine biopsy performed in July 2018, revealed hypercellularity for his age with marked proliferation of dysplastic granulopoiesis and megakaryopoiesis, reduced erythropoiesis and the presence of dense mast cell aggregates with atypical spindle‐shape. The blast count was not increased. BM fibrosis was evident (MF‐2). SM was diagnosed (major criterion and four minor criteria) (Figure 1). The diagnostic criteria of CMML were additionally met (all five criteria) and fittingly, the diagnosis of SM‐AHN was assumed. Cytogenetic testing revealed a normal male karyotype. The molecular analysis of the initial BM sample revealed *KIT* D816V mutation with 90% VAF (variant allele frequency) (Realtime PCR, cDNA). Next Generation Sequencing (NGS) showed positivity for *ASXL1* with 50%VAF, *SRSF2* with 37% VAF, *TET2* with 100% VAF as well as the known *KIT* D816V mutation with a VAF of 46%. For *KIT* D816V, the differences observed between the results obtained by quantitative PCR and NGS were explained by the different starting material, genomic DNA material for NGS and RNA for the quantitative PCR. Quantification of a mutation on the basis of RNA depends on the expression of the mutated gene.

**FIGURE 1:**
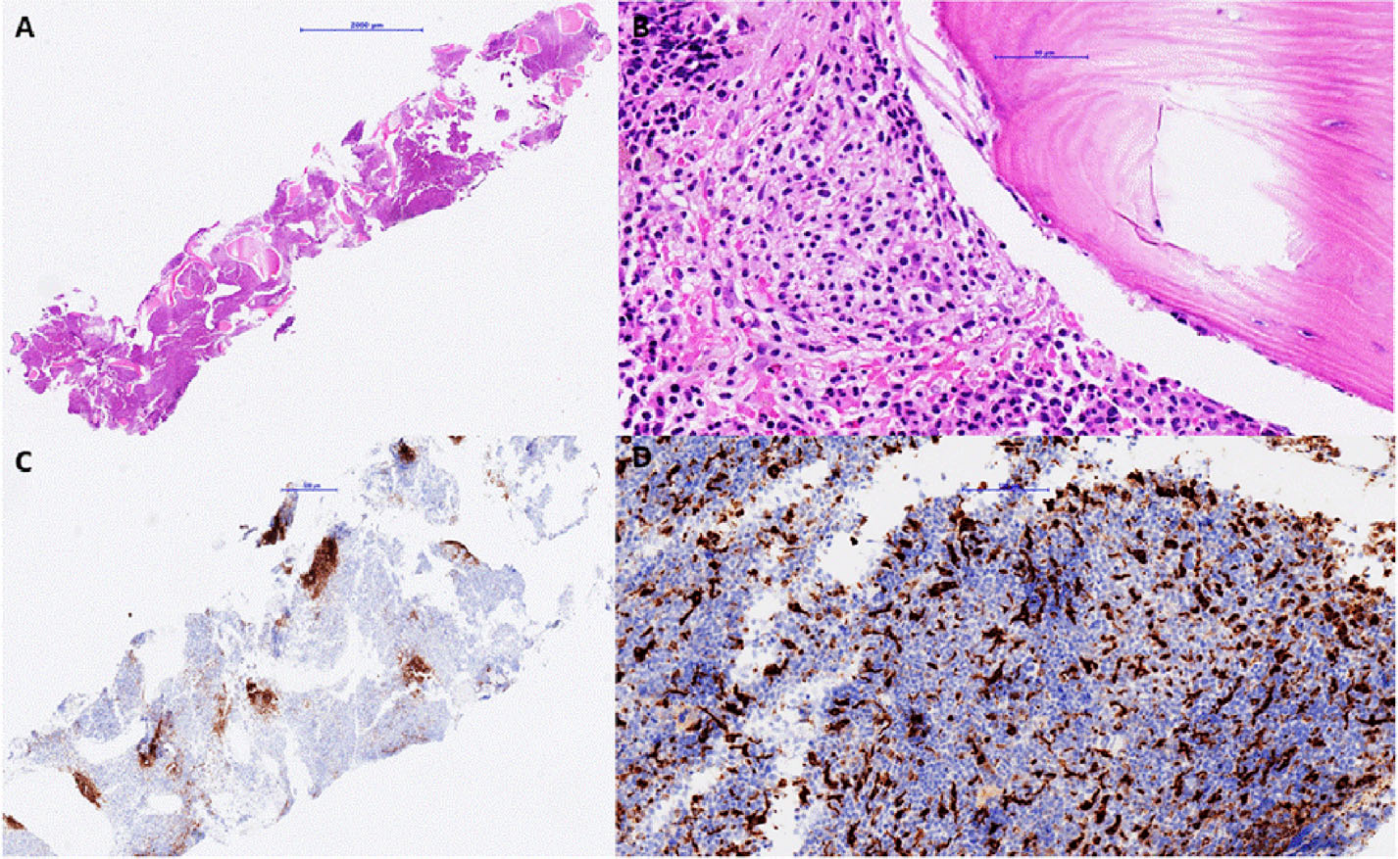
Bone marrow biopsy of the patient. Trephine Biopsy, hematoxylin and eosin (H&E) stain, overview (A) showing a hypercellular bone marrow dominated by multifocal paratrabecular mast cell aggregates (10% of cellularity) and trilinear maturation of haemopoiesis with predominance of the myelod lineage. Detailed image of atypical paratrabecular mast cell aggregates (B). c‐kit immunohistochemistry (C) marks multiple, paratrabecular atypical mast cell aggregates. Increased numbers of macrophages are observed in a CD68 immunohistochemistry (D), showing a slight increase in monocytic cells

Disease evolution was characterized by accelerated progression of hepato‐ and splenomegaly within 4 months (November 2018) while the monocytosis remained stable. We hypothesize that the monocytosis in this case was rather an expression of the underlying mastocytosis and not necessarily proof of evidence of an associated disease as stated in the WHO's diagnostic proposition. We aimed therefore to investigate the molecular characteristics of the monocytes from peripheral blood and compared it with the mutations found in the BM.

Subsequently, in January 2019, in order to separate monocytes from peripheral blood by flow cytometry sorting, we first enriched and isolated peripheral blood mononuclear cells (PBMC) from EDTA‐blood by density centrifugation. PBMC were subsequently stained for 15 min in phosphate‐buffered saline (PBS) using anti‐CD45 V500, anti‐CD14 APC‐H7, and anti‐CD64 APC. PBMC were then washed and resuspended in PBS at a cell density of 10^7^ cells/mL. Samples were analyzed immediately after labeling using a BD FACSAria III instrument. We used Fluorescence activated cell sorting (FACS) method, monocytes were sorted based on FSC^dim^/SSC^dim^ and expression of CD45, CD14, and CD64. Monocyte post‐sorting purity was 97%. At the time of molecular investigations of peripheral blood, on the isolated monocytes and saliva sample, the patient had not received any type of treatment.

For the molecular analysis, only those mutations identified previously in the BM were investigated in the sorted monocytes (Table 1). The monocytes harbored the following mutations: *KIT* *D816V* with VAF of 47%, *ASXL1* with a VAF of 52%, *SRSF2* with a VAF of 53% and *TET2* mutation with a VAF of 100%. Moreover, the high allele burden of *TET2* mutation suggested a germline mutation, so we additionally tested the *TET2* variant p(Asn275Ilefs*18) in a saliva sample using Sanger Sequencing. This was positive with a VAF of 50% suggesting a somatic mutation.

**TABLE 1:** Mutations in the different cell compartments

Mutation	Method	BM	Full blood	Monocytes	Saliva
	Date collected	21 July 2018	22 January 2019	22 January 2019	22 January 2019
		VAF	VAF	VAF	VAF
*KIT D816V*	PCR	90%	43%	47%	–
	NGS	46%	–	–	–
	ddPCR	–	43%	47%	–
*SRSF2*	NGS	37%	47%	53%	–
*ASXL1*	NGS	50%	48%	52%	–
*TET2*	NGS	100%	90%	100%	–
	Sanger sequencing	–	–	–	50%

For NGS, we used the Ion Torrent S5 platform (Thermo Fisher Scientific, Reinach, Switzerland) including IonChef, and the human genome assembly GRCh37 (hg19) for variant calling. The original bone marrow sample was sequenced using the Ampliseq Oncomine myeloid panel (40 genes/hotspots; Thermo Fisher Scientific, Reinach, Switzerland). VAF, variant allele frequency. All molecular investigations were done before the patient received the hypomethylating treatment and also before he underwent an allogeneic hematopoietic stem cell transplantation.

The patient developed a rapidly progressive splenomegaly (26 cm), intrahepatic segmental portal vein thrombosis and extended four‐quadrant ascites after three cycles of hypomethylated therapy. On May 2019, the patient underwent an allogeneic hematopoietic stem cell transplantation from his human leukocyte antigen genoidentical brother. To date, the patient is alive, however without achieving hematologic remission, and has extensive cutaneous graft‐versus‐host disease.

The definition of the role of monocytosis in the diagnosis of myeloid diseases has been topic of discussion for a long time. Monocytosis may accompany many myeloid diseases; the latest WHO update considered this aspect particularly for the diagnosis of CMML, defining that monocytosis not only has to be greater than 1.0 × 10^9^/L, but also has to exceed 10% of the total leukocytes count [1]. This new cutoff should certainly contribute to a better differentiation of many myeloid entities; however, it remains arbitrary and may not be valid in all cases. The identification of *BCR‐ABL1, PDGFRA, PDGFRB FGFR1 PCM1‐JAK2* mutations together with the criteria for the diagnosis of specific diseases like chronic myeloid leukemia, primary myelofibrosis, polycythemia vera, and essential thrombocythemia will rule out the diagnosis of CMML. In all these diseases, monocytosis per se as a criterion is therefore overruled. Mastocytosis is currently not included in this consideration.

Some authors described that multilineage *KIT* involvement and multi‐mutated clones are characteristic of advanced SM, and mentioned that this is particularly true when associated with hematologic neoplasms [2, 3, 4]. The presence of *KIT* D816V was first reported in monocytic cell of BM [2] and a further publication also identified *KIT* D816V mutation in variable myeloid subtypes of marrow cells of AHN [3]. Among multilineage SM patients, the presence of the *KIT* D816V mutation is typically detected in genomic DNA (gDNA) of CD34^+^ hematopoietic stem and precursor cells, eosinophils, monocytes, and maturing neutrophils, and, to a less extent, also in T lymphocytes, in addition to bone marrow mast cells [5]. Using highly sensitive PCR technique, *KIT* D816V mutation can be found in peripheral blood of adult SM patients [6]. A recent a paper revisited the issues related to CMML diagnosis and prognostication in which SM with concomitant CMML is considered as a separate entity within the special variants of CMML (SM‐CMML). This international consensus group highlighted as a key diagnostic feature to discriminate the variant from classical CMML, the presence of *KIT* D816V in most of CMML monocytes 7]. We did not find previous data reporting on the molecular profile beyond *KIT* D816V of isolated monocytes from peripheral blood of SM‐AHN patients.

Historically, monocytosis has proven to be a great confounder. The advances in molecular diagnostics and the improvement of the diagnostic criteria for myeloid neoplasms in the past two decades have contributed to delimiting some myeloid neoplasms even with noticeable monocytosis from CMML [1, 7, 8, 9]. In the case presented, the disease that drove the clinical picture was clearly the SM. Formally, the persistent monocytosis allowed the diagnosis of CMML. The confirmation that the isolated monocyte harbored all mutations found in BM inclusive *KIT* (Table 1) suggests that the monocytes might be rather the expression of the SM and may perhaps not represent a second disease. Associating other diseases with systemic mastocytosis can be a distracting factor to focus treatment properly [10]. We consider that a most appropriate nomenclature for such cases needs to be defined.

## AUTHOR CONTRIBUTIONS

LN and AR contributed to the writing of the manuscript and creation of the table. NP contributes with molecular analysis. MF contributed with the flow cytometry sorting. UB contributed with molecular analysis and diagnostic. YB contributed with the histological diagnosis and creation of the figure. All authors reviewed and approved the final version of this manuscript.

## CONFLICT OF INTEREST

The authors have declared no conflict of interest.
